# Frequency of dendritiform inflammatory cells in the cornea in herpetic anterior uveitis without clinical keratitis and Fuchs uveitis

**DOI:** 10.1186/s12348-014-0031-y

**Published:** 2014-12-31

**Authors:** Alexandra B Knoll, Andreea S Postole, Gerd U Auffarth, Friederike Mackensen

**Affiliations:** 1 Interdisciplinary Uveitis Center, Department of Ophthalmology, University Hospital Heidelberg, Im Neuenheimer Feld 400, Heidelberg, 69120, Germany

**Keywords:** Herpetic anterior uveitis, Rostock Cornea Module, Dendritiform inflammatory cells, Anterior uveitis

## Abstract

**Background:**

Herpetic anterior uveitis is a frequent cause of infectious uveitis. A definite diagnosis is obtained by anterior chamber puncture and polymerase chain reaction, an invasive procedure. We hypothesized that patients with herpetic anterior uveitis have a certain pattern of inflammatory cells in their cornea that distinguishes herpetic anterior uveitis from other uveitis types. This study is a prospective, controlled, observational study. Ten patients are with active herpetic anterior uveitis and 14 patients are with Fuchs uveitis syndrome. Patients were imaged with the Heidelberg Retina Tomograph with the Rostock Cornea Module attachment. Three images of the subepithelial area of the cornea were evaluated for dendritiform inflammatory cells. Means were calculated and used for analysis. The contralateral unaffected eyes and numbers published in the literature served as controls.

**Results:**

The number of dendritiform inflammatory cells in herpetic anterior uveitis was compared to that in the Fuchs uveitis syndrome. Of the eyes of patients with herpetic anterior uveitis, 80% had an average of 98.0±10.8 cells/mm^2^ (mean±standard error of the mean (SEM), *n*=10) in their affected eyes and 60.4±26.4 cells/mm^2^, (*n*=6) in 30% of their fellow eyes. Patients with Fuchs uveitis syndrome had moderately elevated dendritiform inflammatory cells (47.0±9.7 cells/mm^2^, *n*=14) in 96.4% of their affected eyes and normal numbers (23.0±7.3 cells/mm^2^, *n*=13) in 46.4% of their fellow eyes. The difference between the four groups was significant (*p*=0.0004).

**Conclusions:**

Patients with herpetic anterior uveitis had significantly higher levels of dendritiform inflammatory cells in their subepithelial cornea than patients with Fuchs uveitis syndrome, which can be detected by *in vivo* confocal microscopy. The clinically unaffected eyes of herpetic anterior uveitis patients showed a co-response regarding dendritiform inflammatory cell elevation. We conclude that high numbers of dendritiform inflammatory cells in the cornea of uveitis patients may support the clinical diagnosis of herpetic anterior uveitis.

## Introduction

Anterior uveitis (AU) is the most frequent localization of uveitis [1],[2]. Most cases of AU are associated with either a systemic disease (approx. 30%), a clearly defined ocular syndrome (approx. 30%), are unclassified or idiopathic disease (approx. 25%) or with an infection (9.5%). Among the infectious forms, herpes virus infections and Fuchs uveitis syndrome (FUS) are the most common etiologies [1].

The clinical presentations of these two frequent types of AU show similarities, making it sometimes difficult to distinguish them from each other. Both typically present with a unilateral uveitis associated with iris atrophy and intraocular pressure elevation [3] (reviewed in [4]). FUS is more often a chronic low-grade AU with stellate keratic precipitates (KP), and herpetic anterior uveitis (HAU) more often shows a more acute presentation with granulomatous KP, but many cases at first diagnosis show moderate inflammation and mixed KP and only a longer evaluation period shows the differences. An anterior chamber puncture and aqueous humour analysis for viral polymerase chain reaction (PCR) or intraocular antibody synthesis can verify the diagnosis, but patients often prefer to avoid this invasive procedure.

The Heidelberg Retina Tomograph III with the Rostock Cornea Module attachment (HRT-RCM), Heidelberg Engineering, Germany, is a new generation confocal laser scanning microscope which allows corneal imaging on a cellular level. Acquiring those images is non-invasive and fast and bears minimal risk for the patient.

Dendritiform inflammatory cells (DCs) in the subepithelial region (basal epithelium/Bowman's layer or interspersed in the subbasal nerve plexus) can be displayed with this method and appear as large and hyperreflective, branched structures. For easier reading, we decided to refer to these cells as subepithelial throughout the paper even though they may branch into adjacent regions. These cells can be seen by *in vivo* confocal microscopy in up to 30% of healthy corneas in low numbers, with increasing density towards the limbal cornea [5]-[8]. During corneal inflammation (e.g., herpetic keratitis) and irritation (e.g., contact lens wear), increased numbers in the central cornea have been described [5],[8],[9]. To our knowledge, so far, this observation has not been published in non-corneal disease such as anterior uveitis. While we examined corneas of uveitis patients with the HRT-RCM in a previous study looking at keratic precipitates [10], it became apparent that patients with herpetic AU, but not with other types of AU, frequently had high amounts of DCs in their central cornea. We hypothesized that an increase of these cells is typical for herpetic AU and can therefore help to distinguish this type of AU from other AU etiologies.

## Methods

Consecutive patients were prospectively recruited from the clinic of a university-based tertiary centre, the Interdisciplinary Uveitis Centre Heidelberg, Germany. The study protocol was approved by the Institution's Ethical Review Commission. Informed consent was obtained from all patients. The tenets of the Declaration of Helsinki were followed.

Diagnosis of HAU was based on the clinical presentation [11] (unilateral, granulomatous anterior uveitis with intraocular pressure (IOD) elevation [12] and iris atrophy but no corneal involvement) and confirmed either by anterior chamber puncture and PCR (2/10 patients) or by improvement due to acyclovir therapy (10/10). HAU was assumed to be herpes simplex virus AU (five patients), confirmed by aqueous humour analysis in two cases (herpes simplex virus (HSV)-1), or varicella-zoster virus AU (five patients). Two patients had AU for the first time; eight had a recurrent episode of HAU. None of the patients received antiviral therapy at the time of HRT-RCM examination.

Patients who had been diagnosed with FUS were selected from our database and called in for the examination with the HRT-RCM. Diagnosis of FUS was based on a chronic AU with stellate and/or diffuse keratic precipitates, diffuse iris transillumination defects and/or heterochromia which shows constant low-grade iridocyclitis with only little or moderate symptoms and does not improve with any medical treatment [13]. It is therefore possible to plan their examination; an acute flare is not required. None of the FUS patients was on active medications as, for example, local corticosteroids at the time of examination. Aqueous humour analysis had been performed on three FUS patients and showed local anti-rubella virus IgG production (elevated Goldmann-Witmer coefficient) in all cases.

Generally, patients did not undergo ocular surgery in the year previous to the RCM exam.

The patients received an anaesthetic eye drop in both eyes and a drop of an aqueous gel tear substitute (e.g., Vidisic® gel) to optically couple the microscope lens and the cornea. The objective of the microscope was an immersion lens (Olympus, Hamburg, Germany), magnification ×60, to which a disposable contact plastic cap (‘TomoCap’, Heidelberg Engineering, Heidelberg, Germany) was attached. The patients were asked to look straight at a marked point in a 5-m distance, in order to standardize the examination conditions and to scan a corresponding corneal area in all patients as good as possible. HRT-RCM (mag ×400/Achroplan × 63W/NA 0.95/AA 2.00 mm 670 nm/Zeiss, Oberkochen, Germany) scanning was then performed. The focus was manoeuvred through the corneal epithelium along the *z*-axis until the subbasal nerve plexus became visible at a depth of 40 to 60 μm. Sequential images were captured on this level from central areas of the cornea. The scans captured an area of 400 × 400 μm (384 × 384 pixels) per image, with a transverse optical resolution of 2 μm and longitudinal optical resolution of 4 μm (Heidelberg Engineering supplied information). Usually, an exam takes 2 to 5 min resulting in 30 to 100 images of the region of interest.

Two observers (AK and SP), one of them blinded (SP) to the patients' diagnosis, selected three representative images per patient and counted the number of cells per square millimetre using the system's cell counting software as described in [7]. DCs touching two of the four edges of the image were excluded to avoid under/overestimation of the density. Manual lateral scanning was used to trim and image the nerve plexus in the central region of the cornea. Means of the six countings were calculated and used for analysis. Published numbers of DCs in healthy central corneas at a depth of 35 to 60 μm, 34 ± 3 cells/mm^2^ (range 0 to 64 cells/mm^2^), served as normal controls [7]. The methodology of this study is comparable to our study. GraphPad Prism Software was used for statistical analysis. Results were evaluated for statistical significance with a Mann-Whitney *U* Test or an ANOVA when appropriate.

The interobserver reliability was calculated with a Spearman test performed on the mean cells per square millimetre per patient by each observer.

## Results

Ten patients with active HAU (10 active and 6 contralateral unaffected eyes) and 14 patients with FUS (15 affected and 13 unaffected eyes, 1 patient had bilateral, albeit asymmetric (OS > OD), Fuchs) were examined with the HRT-RCM between 2009 and 2012. Five HAU patients were female, and five were male, mean age at examination was 50.3 years (range 15 to 70 years). Five FUS patients were female and nine were male, mean age was 49.8 years (range 30 to 69 years). None of the patients with HAU showed signs of corneal affection on slit lamp examination. Corneal sensitivity was tested with esthesiometer in five of the HAU patients and was markedly reduced in two, slightly reduced in two, and normal in one patient. The remaining HAU patients were not tested.

Interobserver correlation of DC counts was excellent (Spearman *r* = 0.9622, *p* ≤ 0.0001).

**Figure 1:**
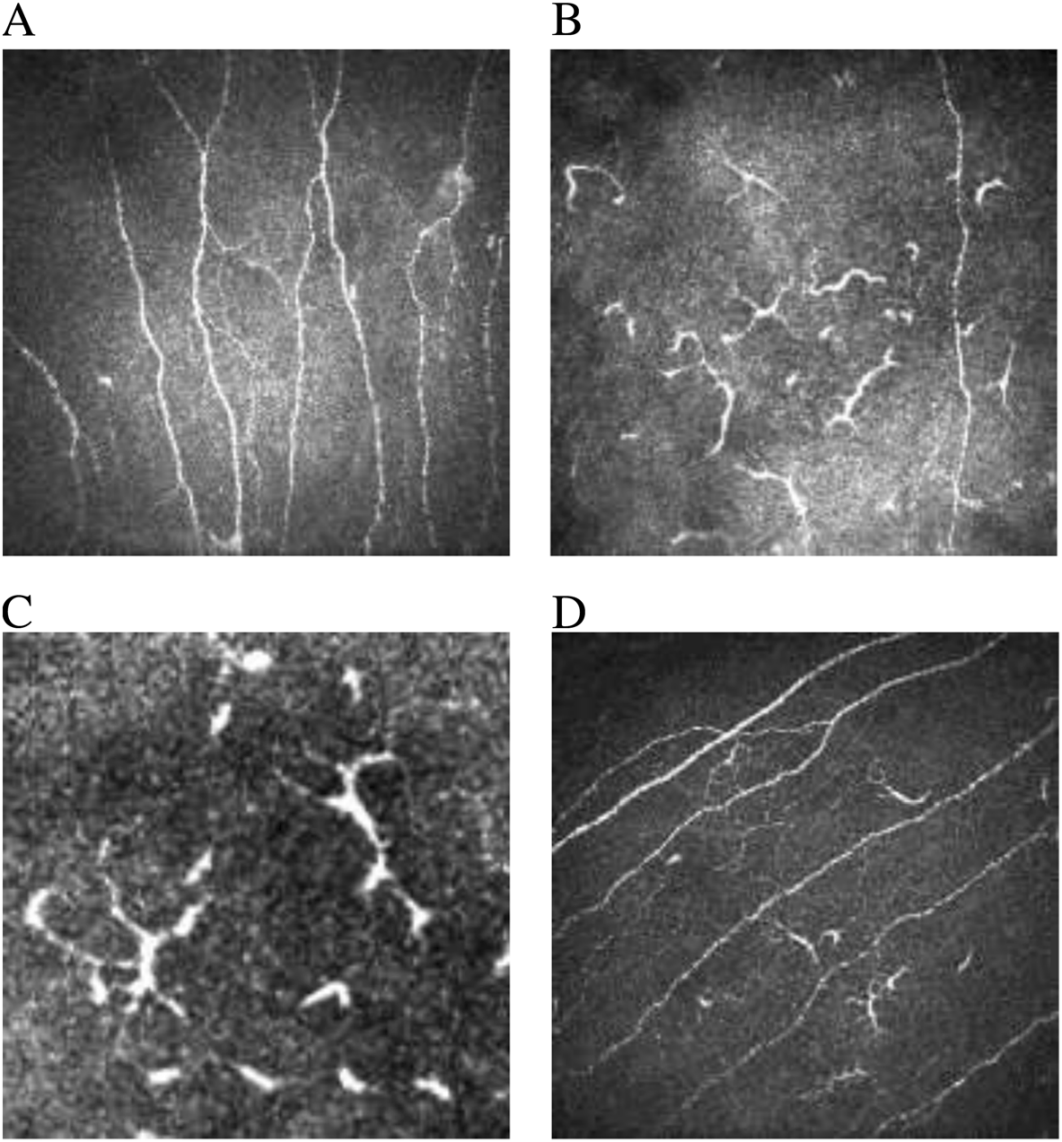
**Dendritiform cells (DCs) display.** As large, hyperreflective, branched structures in the subepithelial region of the cornea above of or interspersed in the subbasal nerve plexus. Bar represents 50 μm. **(A)** The corneal subbasal nerve plexus in a healthy cornea (400 × 400μm). **(B)** DCs in one section (400 × 400 μm) in the cornea of a patient with herpetic anterior uveitis. **(C)** Close up of DCs. **(D)** Subbasal nerve plexus of a patient with FUS showing some DCs.

**Figure 2:**
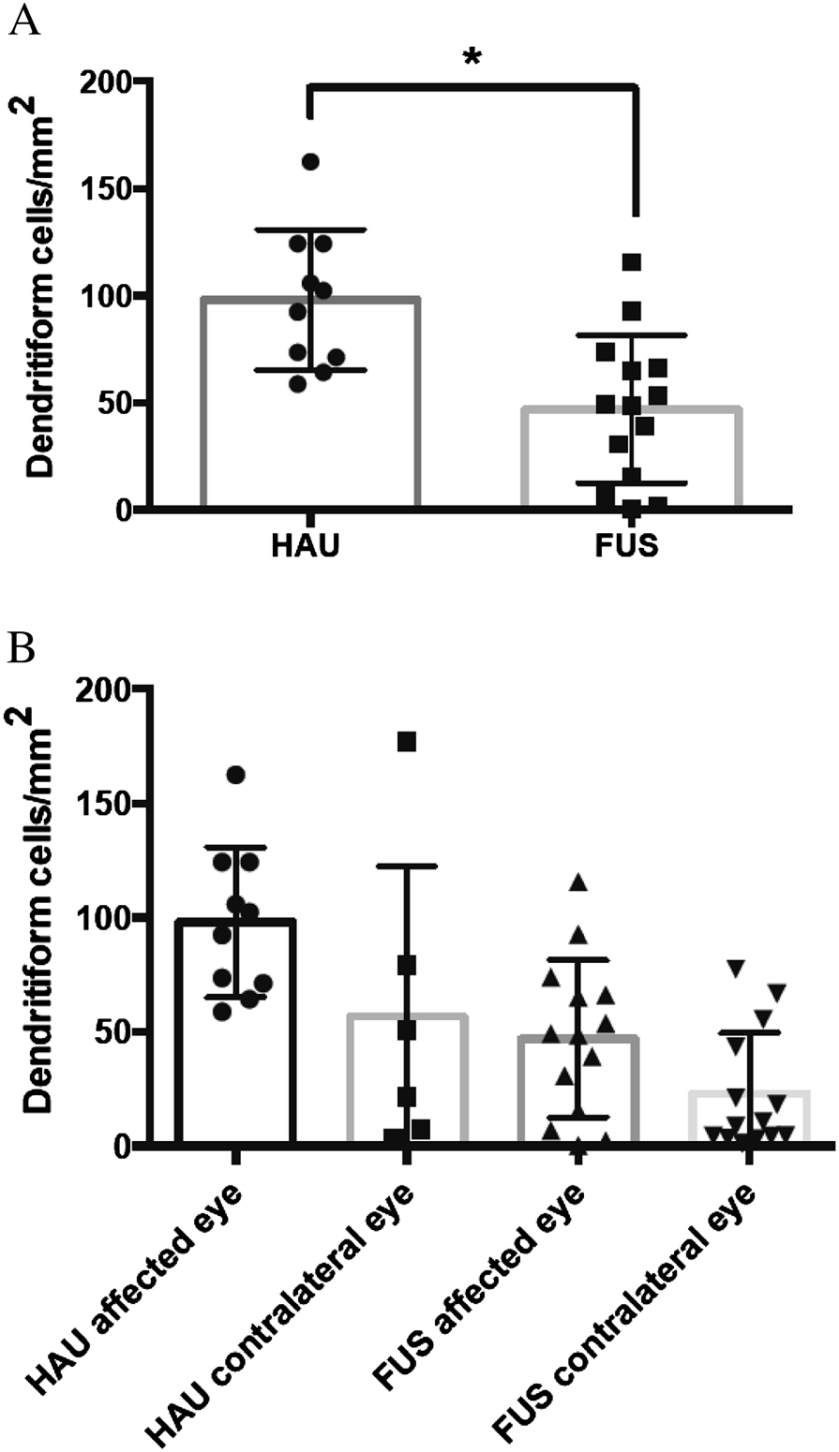
**Amount of dendritiform cells (DCs) per square millimetre.** In the subepithelial corneas of patients with herpetic anterior uveitis (HAU) vs. Fuchs uveitis syndrome. **(A)** Significantly, more DCs were present in the affected eyes of HAU compared with FUS (*p < 0.0001 Mann Whitney *U* test). **(B)** Both eyes of patients with herpetic uveitis showed increased DCs in their corneas, indicating a co-response of the fellow eye. DCs in the affected eyes of FUS patients were moderately elevated but not in the unaffected eyes.

In both groups, DCs were seen in higher frequency in the subepithelial area of central corneas of the affected eyes compared to the fellow eyes (Figure 1). We saw DCs in all affected HAU eyes and five of six (83%) fellow eyes. In FUS patients, DCs were seen in 12 of 14 (86%) affected and 12 of 13 (92%) fellow eyes. Patients with HAU had significantly more DCs (98.0 ± 10.8 cells/mm^2^, mean ± standard error of the mean (SEM), range 58.8 to 162.6 cells/mm^2^) in their affected eye than patients with FUS (47.0 ± 9.7 cells/mm^2^, mean ± SEM, range 0 to 115.6 cells/mm^2^, *p* < 0.0001) (Figure 2A). Patients with HAU also showed elevated numbers of DCs (60.4 ± 26.4 cells/mm^2^, range 0 to 177.3 cells/mm^2^) in their contralateral, clinically unaffected eye. Contralateral eyes of FUS patients exhibited only few DCs (23.0 ± 7.3 cells/mm^2^, range 0 to 77.5 cells/mm^2^), lower than numbers found in healthy corneas (34 ± 3 cells/mm^2^ (range 0 to 64 cells/mm^2^) [7] (Figure 2B). One patient with bilateral FUS showed a mean of 45 cells/mm^2^ DCs in the right, less affected eye and 59 cells/mm^2^ DCs in his left, more affected eye.

The amount of DCs did not correlate with the anterior chamber cell count or anterior chamber flare, assessed by slit lamp examination (data not shown). Anterior chamber cells ranged between 0 and 2+ (2+ only in one patient) in the HAU group and between 0 and 1+ in the FUS group.

## Discussion

Laser *in vivo* confocal microscopy has been used over the past years by different investigators to image corneal sections [6],[7],[9],[14]-[16] for a review [8]. Hyperreflective cells of dendritic appearance have frequently been described in the basal epithelium or subepithelial region at the level of Bowman's layer and sometimes are referred to as Langerhans cells or dendritic cells [7]. Studies performing immunohistological staining on animals [14],[17] and a recent study on humans plausibly suggest that cells with branching, dendritic morphology in the corneal basal epithelial layer are antigen-presenting cells [18]. Most likely, a small heterogeneous population of antigen-presenting cells is present in uninflamed corneas which undergo changes in phenotype and function during corneal inflammation [6],[17]-[20].

In 1987, an animal study showed a significant increase of Langerhans cells in the central cornea during HSV-1 keratitis, assessed by cytochemistry [21]. A more recent study on mice suggested an essential role of corneal dendritic cells in the immune defence against HSV-1 keratitis, by directing the local NK response [22]. Mayer et al. found more antigen-presenting cells in post-herpes keratitis corneas than in graft rejection after keratoplasty or keratoconus, assessed via confocal microscopy and histochemistry [18]. Mocan et al. found dendritiform and small round cells by *in vivo* confocal microscopy in 52% of patients with non-epithelial herpes keratitis. This included eight patients with keratouveitis, which showed DCs in 38%. In their healthy controls, these authors apparently did not see any DCs [9]. Rosenberg et al. found DCs in 62.5% of eyes with HSV keratitis and 12.5% of fellow eyes [5]. This is in contrast to our study where DCs were seen in 86% to 100% of affected and fellow eyes and also to another study on patients with herpetic keratitis reporting DCs in 92% of affected and 21% of healthy control eyes [6]. This difference in DCs detected may be explained by the confocal microscope used, Mocan et al. using a slit scanning confocal microscope (Confoscan 3.0), Rosenberg et al. a tandem scanning confocal microscope, and we and Mastropasqua as well as Zhivov et al. used a laser scanning confocal microscope (HRT-RCM) [6],[7].

Regarding mean numbers of DCs detected, our numbers are lower than those found in HSV keratitis (241.9 ± 81.1 cells/mm^2^) or corneal graft rejection (147.2 ± 32.5 cells/mm^2^) by Mastropasqua et al. This could be explained by secondary or collateral involvement of the cornea in HAU, the primary herpetic reactivation being in the ciliary nerves of the iris.

To our knowledge, no studies have been published on the behaviour of dendritiform cells during anterior uveitis without corneal involvement.

We found that in patients with herpetic AU, despite no clinical signs of keratitis, the central corneas contained high numbers of DCs. In our control group with FUS, a clinically similar uveitis form supposedly of viral origin (Rubella) as well [23],[24], the number of DCs was only moderately increased with regard to the average measured in healthy probands [6],[7]. Therefore, we see a potential diagnostic value for this method in diagnosing HAU non-invasively, but other non-infectious anterior uveitis subsets should be evaluated to create cutoff values for DC counts that confirm a diagnosis of HAU.

Elevated DCs even in the contralateral, uninflamed eye of patients HAU indicated a co-response of the fellow eye. This is in keeping with recent results by Hamrah et al. who found a nerve fibre reduction in both eyes of patients with unilateral zoster ophthalmicus keratitis, similarly suggesting bilateral changes in a clinically unilateral herpetic eye disease [25]. Neither the study of Mocan et al. nor the study of Mastropasqua et al. did look at DC numbers in the contralateral eye [6],[9]; Rosenberg et al. saw DCs in 12% of fellow eyes but did not quantify them [5].

Dendritic cells, which probably compose at least in parts the DCs we here describe, might appear for immunological reasons in the cornea while the herpes virus affects not the cornea itself but the adjacent anterior chamber. Herpetic keratitis has been studied more intensely than herpetic uveitis, but the exact pathogenesis remains insufficiently understood [26]. After primary infection, the herpes virus passes into a state of latency (intact quiescent viral genome without obvious pathological effects) in a host neuronal ganglion cell until its reactivation (migration to the site of infection and replication), often triggered by stress (reviewed in [27]). Not only the trigeminal ganglion is generally accepted to be a site of HSV latency [28],[29], but also the ciliary ganglion has been found to host HSV DNA in asymptomatic humans, offering possible explanations for anterior chamber or retinal herpetic diseases, tissues not directly innervated by trigeminal nerves [30]. A topic of great debate is whether the cornea itself may be a reservoir for the inactive herpes virus [31],[32]. Some manifestations of herpetic keratitis, particularly herpetic stromal disease, are assumed to be virus-independent and rather than immune-mediated diseases. Over 20 years ago, studies showed that an increase of Langerhans cells in the cornea prior to infection with HSV-1 led to a significantly higher susceptibility and severity of herpetic stromal disease [33],[34].

There are limitations to our study. We - as other studies - can only present a small number of patients, which lies in the nature of the disease. Still, we present two homogenous uveitis subtypes. Unfortunately, we could only give molecular proof of viral infection in two of the HAU patients, as the remaining patients declined anterior chamber tap. In the best of possible cases, we would not only have done PCR to show viral DNA but also tested for local antibody production. By combining PCR, viral load and local antibody production, a better correlation with the level of viral replication and antiviral immunity could have been achieved [3],[35]. Equally, our choice of FUS as a control could be discussed as not ideal, as it is a chronic disease, with only mild inflammatory activity. In our study, the amount of corneal DCs did not correlate with the anterior chamber cell count or flare. We therefore believe that their increase does not reflect increasing intensity of unspecific inflammation but possibly indicates active immune mechanisms against the herpes viruses. Therefore, we also think that the differences in numbers of DCs seen between HAU and FUS are not due to less inflammatory activity in FUS. Still, to further support this theory, DCs in patients with other forms of AU need to be studied. Further, this is a cross-sectional study; no serial confocal microscopic exams were performed. Lastly, we only collected information on the central cornea. However, as shown in the study from Mastropasqua et al., this is the region of interest for corneal inflammatory diseases, showing the most significant differences towards healthy controls [6]. Other authors reported a decreased subepithelial nerve fibre density in the central corneas in herpetic keratitis as an additional finding [9],[36]. We have not looked into this in the study presented here. The HRT-RCM is a non-invasive and low-risk technique to acquire corneal images. DCs are easily displayed, and high amounts were seen in our study in patients with HAU. We conclude that confocal microscopy may be a useful additional tool in diagnosing HAU. How DCs behave in quiescent or treated disease as well as in other anterior uveitis subtypes remains to be evaluated.

## Conclusions

We conclude that confocal microscopy may be a useful additional tool in diagnosing HAU. How DCs behave in quiescent or treated disease as well as in other anterior uveitis subtypes remains to be evaluated.

## Abbreviations

AU: anterior uveitis

DC: dendritiform inflammatory cells

DNA: desoxyribonucleic acid

FUS: Fuchs uveitis syndrome

HAU: herpetic anterior uveitis

HRT: Heidelberg Retina Tomograph

HSV: herpes simplex virus

IOD: intraocular eye pressure

KP: keratic precipitates

OD: oculus dexter

OS: oculus sinister

PCR: polymerase chain reaction

RCM: Rostock cornea module

SEM: standard error of the mean

## Competing interests

Friederike Mackensen has received lecture honoraria by Heidelberg Engineering, and Alexandra Knoll has received a Travel Grant by Heidelberg Engineering. We have been loaned the Heidelberg Retina Tomograph III and the Rostock Cornea Module attachement (HRTIII RCM) by Heidelberg Engineering for this study and another study. The other authors declare that they have no competing interests.

## Authors' contributions

ABK made substantial contribution to the acquisition of data and analysis and interpretation of data and drafted the manuscript. ASP made substantial contribution to the acquisition of data, participated in its coordination and helped to draft the manuscript. GUA had been involved in the design and coordination of the manuscript and helped to draft the manuscript. FM participated in its design and coordination, revised the manuscript critically for important intellectual content and agreed to be accountable for all aspects of the work in ensuring that questions related to the accuracy or integrity of any part of the work are appropriately investigated and resolved. All authors read and approved the final manuscript.
